# Simulating institutional heterogeneity in sustainability science

**DOI:** 10.1073/pnas.2215674121

**Published:** 2024-02-15

**Authors:** Michael R. Davidson, Tatiana Filatova, Wei Peng, Liz Verbeek, Fikri Kucuksayacigil

**Affiliations:** ^a^School of Global Policy and Strategy, University of California San Diego, La Jolla, CA 92093; ^b^Department of Mechanical and Aerospace Engineering, University of California San Diego, La Jolla, CA 92093; ^c^Department of Multi Actor Systems, Faculty of Technology, Policy and Management, Delft University of Technology, 2628 BX Delft, The Netherlands; ^d^School of Public and International Affairs, Princeton University, Princeton, NJ 08544; ^e^Andlinger Center for Energy and the Environment, Princeton University, Princeton, NJ 08544

**Keywords:** sustainability modeling, institutions, integrated assessment, optimization, agent-based model

## Abstract

Sustainability outcomes are influenced by the laws and configurations of natural and engineered systems as well as activities in socio-economic systems. An important subset of human activity is the creation and implementation of institutions, formal and informal rules shaping a wide range of human behavior. Understanding these rules and codifying them in computational models can provide important missing insights into why systems function the way they do (static) as well as the pace and structure of transitions required to improve sustainability (dynamic). Here, we conduct a comparative synthesis of three modeling approaches— integrated assessment modeling, engineering–economic optimization, and agent-based modeling—with underexplored potential to represent institutions. We first perform modeling experiments on climate mitigation systems that represent specific aspects of heterogeneous institutions, including formal policies and institutional coordination, and informal attitudes and norms. We find measurable but uneven aggregate impacts, while more politically meaningful distributional impacts are large across various actors. Our results show that omitting institutions can influence the costs of climate mitigation and miss opportunities to leverage institutional forces to speed up emissions reduction. These experiments allow us to explore the capacity of each modeling approach to represent insitutions and to lay out a vision for the next frontier of endogenizing institutional change in sustainability science models. To bridge the gap between modeling, theories, and empirical evidence on social institutions, this research agenda calls for joint efforts between sustainability modelers who wish to explore and incorporate institutional detail, and social scientists studying the socio-political and economic foundations for sustainability transitions.

## Significance

Sustainability outcomes are influenced by natural and engineered systems, as well as by social institutions—rules and norms in socio-economic systems. While the importance of formal and informal institutions is well established, incorporating institutions into computational models is challenging, limited by model structure and availability of relevant datasets. We compare simulation outcomes of three approaches: integrated assessment modeling, engineering–economic optimization, and agent-based modeling. Through adding institutional factors, we demonstrate concrete ways sustainability models can be enhanced to address real-world questions such as consumer adoption of clean energy technologies and national costs of climate mitigation. The next modeling frontier is to include how institutions evolve over time toward sustainability transitions, calling for joint efforts between modelers and social scientists.

Achieving sustainability outcomes through socio-technical transitions depends on an understanding of heterogeneous nature–society interactions which requires combining insights across disciplines and epistemological traditions (1, 2)**.** Computationally, significant advances have been made to model sustainability outcomes, including physical processes (e.g., Earth system models), engineered systems related to advancing sustainability (e.g., multi-energy sector models), economy–energy interactions of human activities (e.g., integrated assessment models) (3, 4), and bounded rationality and social networks (e.g. agent-based models) (5). However, across the field, there are inconsistent and incomplete characterizations in sustainability models of *institutions*—the formal and informal rules constraining human behavior (6). As the rules defining socio-economic dynamics, institutions are crucial to understanding current sustainability crises due to their wide-ranging effects on the actions of humans and groups of individuals (7, 8). By extension, institutional change is essential to addressing complex sustainability challenges such as energy transitions (9). To date, due to their ease of implementation and interpretation in computational sustainability models, institutions that are represented in simulations typically consist of simplified policies such as emissions targets and changes in technology costs (via taxes or subsidies), which are only a subset of formal or parchment institutions**.** By contrast, modeling is scarce for the broader range of formal institutions (e.g., policy mechanisms, policy processes, and interactions at the interfaces of different political and economic institutions) as well as informal institutions (e.g., norms and socio-cognitive beliefs)**.** This broader set of institutional factors can shape human behavior and nature–society systems, resulting in deviations from typical welfare-maximizing or least-cost model solutions that may not be feasible to implement in the real world (10, 11). However, the modeling literature rarely scrutinizes these institutional “rules of the game.” The implications for sustainability science and sustainability policy are manifold, ranging from inaccurate descriptions of complex interactions shaping today’s sustainability outcomes (including adaptive human behavior) to unrealistic prescriptions for improving future sustainability trajectories (1, 12).

Computational models provide an opportunity to widen our methodological approach to study the role of institutions on sustainability, complementing and building on voluminous social science traditions in institutional economics, historical institutionalism, rational choice theories of institutions, and sociological or organizational behavior (6, 13–17). Currently at the center of computational models of the nature–society interface is a techno-economic representation, which is the combination of technological constraints and economic incentives that generate real-world trade-offs in sustainability-relevant sectors such as energy and land use (9). Institutions, to a first degree, layer on additional constraints and incentives, which must be considered in concert with natural processes and techno-economic criteria to understand the dynamical properties of systems. Besides institutions, there are other factors in the blind spots of sustainability models, such as adaptive behavior, imperfect information and learning, and risk aversion (18). Still, many of these socio-behavioral processes relevant to sustainability transitions cannot be fully understood without scrutinizing the role of institutions.

For instance, models can simulate varying levels of individual incentives to take action on sustainability, which are affected by social norms and beliefs (equivalently, modifying our assessment of welfare functions to include non-economic considerations). Models can also estimate the redistribution of costs and benefits of transitions, providing insights into which segments of society become winners or losers, or how interest groups or political coalitions may evolve in size, power, and policy-making influence. Furthermore, models can explore to what extent and which informal institutions such as normative or socio-cognitive beliefs could hinder or propel, respectively, individual actions that are otherwise economically optimal (19). These are important questions because institutions mediate the behavior of key actors (e.g., firms, households); they also shape and are shaped by interest groups (e.g., industries, civil society organizations) mobilizing to defend their priorities and influence the creation of new rules, such as environmental regulations or subsidies, through political channels (20, 21). Fundamentally, achieving sustainability requires societal transformations—the change of incumbent institutions—which is an endogenous process composed of feedback loops (e.g., norms or policies shaping actor behavior which in turn alter the institutional setup). Here, the promise of models is to provide quantitative insights into the strength and speed of different feedback loops, offering insights into the pace and pathways of eventual sustainability transitions.

In this study, with a focus on energy transitions, we evaluate the impacts of inclusion of specific sources of institutional heterogeneity in three modeling methods widely used in sustainability science: integrated assessment modeling (IAM), engineering–economic optimization (EEO) modeling, and agent-based modeling (ABM). To this end, we aspire to answer: How do explicit institutional representations change the aggregate and distributional outcomes of models commonly used in sustainability science? In contrast to attempting a fully endogenous representation of all relevant institutions, we use structured simulation experiments to test out specific institutional factors that are carefully selected based on the structure and logic of each model. Typically, for each model, the parameter settings are systematically varied to represent different versions of reality—for example, highlighting specific representations of modelable institutions. We run structured experiments with each model to answer a specific research question, which in our case explores whether and how adding institutional detail to commonplace sustainability models impacts its core outcomes. We explore endogenous institutional change in one of the experiments (i.e., ABM), highlighting the promise of future work to capture sustainability transitions.

## Representations of Institutions in Three Sustainability Models

### Three Types of Models.

Here, we consider three classes of models that have been used widely in sustainability research^*^: detailed-process IAM, EEO modeling, and ABM. To put a spotlight on how institutions are traditionally represented in these different types of models popular in sustainability science, we keep the spatial scale of analysis at which these models are commonly designed and which are difficult to change without breaking the model essence. This implies that the three models present analysis for different geographical scales: global or national for IAMs, one sector in a regional economy for EEO, and urban areas for ABMs. Below, we provide a short summary of each type of model and their current representations of institutions.

#### Integrated assessment modeling (IAM).

IAMs were initially developed to project global energy and land use emissions of greenhouse gases and the resulting impacts on the global climate. There are two types of IAMs: 1) aggregate, benefit–cost IAMs that use stylized representations to examine optimal emission trajectories that maximize global social welfare, and 2) detailed-process IAMs that have been used to study cost-effective technology pathways to achieve decarbonization goals at regional and global scales (22). While efforts have been made to incorporate representations of socio-political drivers in benefit–cost IAMs (23), those models are too stylized to inform concrete decisions by energy investors and consumers. Therefore, in this paper, we focus only on detailed-process IAMs that have rich representation of technologies and sectors, making it more consistent and comparable with the other two models included in this paper. Detailed-process IAMs typically use exogenous assumptions for the socioeconomic and human system, such as GDP and population growth, as well as policy targets (e.g., 2-degree goal). For instance, the Shared Socio-economic Pathways (SSPs) have been used extensively in climate change assessment (24). The SSPs represent storylines for diverse socioeconomic futures, including institutions and human values, which will likely pose different levels of challenges for climate mitigation and adaptation. Notably, there have been nascent efforts in improving the representations of heterogeneous actors (e.g., urban vs. rural groups) and political economy considerations (e.g., regional variations in institutional quality and policy ambition) in detailed-process IAMs, which involves working closely with social scientists (18, 25–29). These exogenous socioeconomic assumptions provide an avenue to couple with other models, e.g., integrating the demand projections from ABMs that considers heterogeneous consumer behaviors (30).

#### Engineering–economic optimization (EEO) modeling.

EEOs are used for the design, planning, and operations of physical infrastructure systems, such as the electricity grid (31). These models usually include detailed representations of underlying infrastructure (e.g., power plants and transmission lines) as well as the key features and physical rules of the systems (e.g., electricity flows and instantaneous supply–demand balance constraints). Optimization methods are used to identify the investment and operational decisions to achieve certain goals (e.g., least-cost power production). In most EEOs, human demands are exogenously defined based on population characteristics such as socio-demographic conditions as well as climatic conditions (32, 33). Some EEOs also include a more dynamic, endogenous representation of demand, e.g., demands responding to changes in prices or to meet a larger set of welfare objectives (34, 35). While markets are often modeled implicitly in EEOs (e.g., electricity trade between interconnected regions), explicit market rules, institutional frictions across borders, and many other behavioral factors are ignored (i.e., assumed non-existent) in the models. Enhancing policy realism in EEOs, a recent focus of modeling studies, is addressing part of this gap in institution modeling, such as capturing subnational heterogeneity in policy instruments (36), policy coordination (37), and energy development policy constraints on land use (38).

#### Agent-based modeling (ABM).

ABM is a computerized representation of many diverse adaptive boundedly rational actors—e.g., farmers, fishermen, households, firms, governments—that act, interact with each other and the environment, and learn and update their expectations about the future based on the past experience of themselves and others (39, 40). Due to their flexibility in accommodating a variety of assumptions about human behavior, social institutions, and the environment, ABMs are actively applied to study spatio-temporal dynamics of nature–society systems and to explore a wide range of sustainability questions (41). In application to energy transitions specifically, ABMs have a strong track record to model out-of-equilibrium carbon and electricity markets, diffusion of green-energy technologies such as solar panels or electric vehicles, and even climate change negotiations across countries mimicking some features of IAMs (5). ABMs can represent different behavioral theories—beyond the rational perfectly informed optimizer—and hence are instrumental to explicitly incorporate beliefs, attitudes, learning and social norms, permitting direct integration of normative and socio-cognitive institutions in sustainability science models. Since the late 1990s, ABMs also include modeling of formal institutions like markets (42), though they still typically focus on a single market in isolation (albeit increasingly empirically grounded), omitting vital cross-sectoral impacts and technological details on the energy production side. Agent-based computational models of markets often disaggregate supply and demand sides into bilateral interactions of thousands of heterogeneous buyers and sellers with boundedly rational expectations. This permits modelers to study structural shifts in markets, for instance, due to endogenous changes in preferences (43, 44) or technological learning (45), but such models could be computationally expensive to design and scale up.

### Model Representation of Actors and Institutions.

**Fig. 1.**
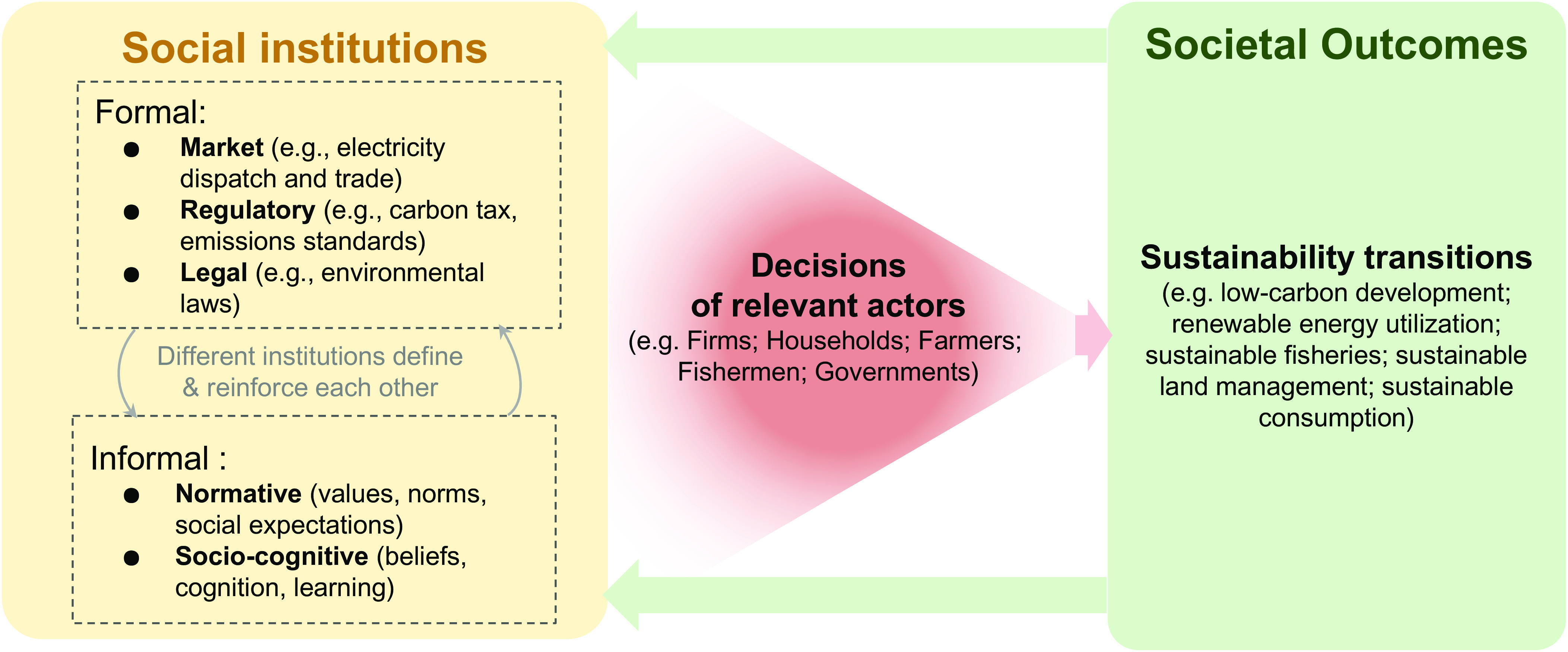
Social institutions, including interactions between formal and informal, affect actors’ decisions in sustainability-relevant sectors, and therefore sustainability transitions. Societal outcomes in turn influence the evolution of social institutions.

Institutions can be conceptualized as the rules that shape the behavior of human or organizational actors, their interactions, and enforcement mechanisms (6, 46). Formal institutions constitute markets, policies, and laws. Informal institutions are typically unwritten rules-in-use shared by a specific society or community, social norms, and other levers around individual decision-making. While informal institutions are shaped, conveyed, and reinforced outside of regulatory, legal, or market dimensions, they could appear very persistent and could define the formal institutions. Further typologies distinguish three categories of institutions: regulative (i.e., formal market and regulatory rules and laws), normative (i.e., informal values, norms, social expectations), and socio-cognitive (i.e., informal beliefs, cognition around decision-making, learning) (19, 47). Both formal and informal institutions shape relevant actors’ decision-making related to broader sustainability transitions like low-carbon development (Fig. 1). Societal outcomes in turn influence the evolution of social institutions (48, 49), implying that, for example, a (lack of) progress on sustainability transitions might spur new market or regulatory institutional arrangements or even a shift to another social norm regarding environmental management.

Formal and informal institution representations must be considered carefully in computational implementations, as models differ in the degree to which they explicitly characterize institutions. Importantly, models employed to study nature–society interactions—the focus of this special feature—already encode some assumptions of institutions, but only a few encode institutions explicitly. Models often make idealized assumptions for formal institutions, including that policy mechanisms are aligned with the regulatory objectives, markets function as expected, and enforcement is often taken for granted. Recent work is beginning to interrogate assumptions of policy enforcement and well-functioning market institutions (28, 29, 50). Assumptions are encoded in models for informal institutions as well, such as rationality, preferences as shaped by social norms, and beliefs, or lack thereof. Sometimes, institutions enter models as scenarios, like policy targets for climate change mitigation. When represented as scenarios, modeled implicitly or explicitly, institutions typically remain static in modern sustainability models.

Parsimoniously separating the effects of formal versus informal institutions in models can be difficult, as they affect and reinforce each other (48, 49). For example, we wish to project the future deployment of renewable energy in Germany. A model-relevant question may involve simulating the evolving level of subsidies provided by renewable energy surcharges—a key incentive driving deployment. However, a persistent, high level of subsidies could be explained by different institutional phenomena. On the one hand, it could result from interest groups (e.g., coalitions of renewable energy industries and universities) entrenching subsidies via growth and capture of political institutions (51). On the other hand, it may also be driven by informal institutions, such as German consumers’ high willingness to pay for green attributes (52). The complex causal mechanisms and the difficulty to empirically quantify their relative contributions make modeling these different institutions a challenging task.

**Fig. 2.**
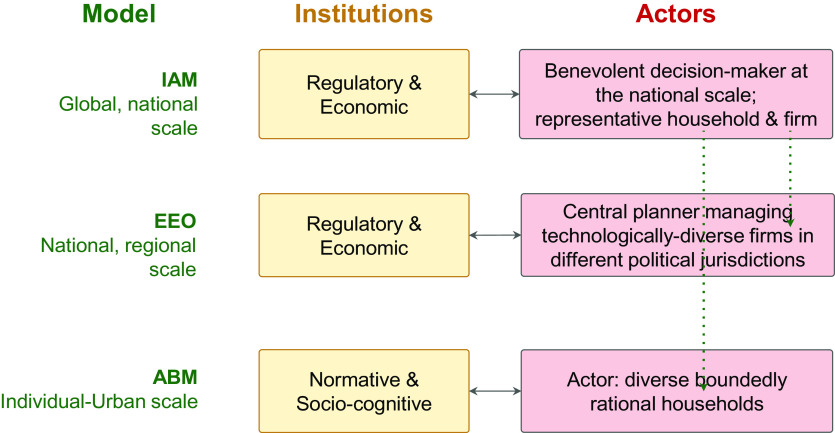
Sustainability models capture different subsets of institutions (regulatory, economic, normative/socio-cognitive) and actors, which are partially shared (indicated by green lines) across the three modeling frameworks.

Looking across the three models considered in this study, they are set up to represent different types and granularity of actors, institutions, and actor-institution interactions (Fig. 2). For instance, IAMs often operate at the global and national scales. They often implicitly assume benevolent decision-makers at the national level and consider representative households and firms. IAMs also implicitly consider regulatory and economic institutions (e.g., national governments). In contrast, regional-scale EEOs include diverse technological interactions (e.g., renewable energy grid integration) and individual generators which could be distinct firms (e.g., coal vs. renewables) interacting with regulatory and economic institutions in different jurisdictions, though the model simplifies firm-level interactions in terms of a perfectly competitive market or, equivalently, single central planner. Finally, individual-scale ABMs consider diverse boundedly rational households who are affected by normative and socio-cognitive institutions (e.g., social norms and behavioral rules).

In this study, we perform concrete simulation experiments using IAM, EEO, and ABM, respectively, to assess the effects of incorporating selected institutions explicitly into sustainability models. We define here three interrelated concepts: A **model** is a software implementation of some conceptualization of reality (e.g., IAM, EEO, and ABM are all models). A **simulation** is a dynamic process of running the model, making it evolve over time (e.g., the results we present are from the simulation runs of our models). A **(simulation) experiment** refers to running the model with specific settings of parameter values and structural model settings to answer a specific research question (e.g., two sets of experiments are Reference vs. Heterogeneous Institutions).

Due to their very different geographic and sectoral contexts, these models have different strengths, and model choice should depend on the research question, desired scope, and available data. To systematically choose institutional factors to represent in the model, we follow the classification put forward in the energy transition literature (47), which in turn relies on the widely accepted differentiation among institutions in sociology (19)^†^. For each model, we formulate two main sets of modeling experiments: 1) “Reference Institutions” represents traditional modeling assumptions of implicit (and often, idealized) institutional representations; 2) “Heterogeneous Institutions” represents modeling configurations where select real-world institutions are made explicit considering respective heterogeneity. Institutions are selected among existing classifications in the energy transition modeling literature, which are predominantly exogenous and recognize models’ respective strengths (47). The main dimensions of heterogeneity explored differ for the three models—policies, institutional coordination, and informal attitudes and norms—reflecting model choice and research question.

Our aim is not to comprehensively consider all heterogeneous institutions relevant to the system, but to shed light on how three common types―formal/regulative, normative, and socio-cognitive institutions (19)—are represented in different types of popular sustainability models. By demonstrating concrete ways that the three models can improve the representation of institutions within the current model structures, our goals are to shed light on the selected institutional considerations, identify the strengths and limitations of each model to represent diverse actors, rules, and norms, and discuss how sustainability science could proceed to better capture institutions in formal models. Our emphasis is also not to compare results between different models. Instead, we assess how heterogeneous institutions are traditionally implemented in commonly used models, using energy transitions as an example, and demonstrate how institutions affect outcomes of each model. This core feature distinguishes our study from a model intercomparison project—where models representing roughly the same things are compared and contrasted to understand what contributes to different results (53).

## Results

To shed light on the conventional representation of institutions in models common in energy transitions (as an example of a sustainability challenge), we quantitatively assess the effects of the three types of institutions—formal/regulative, normative, and socio-cognitive—using our three models. Following the strengths of the IAM, EEO, and ABM models, we test selected institutions from one of the above types. Namely, as an example of a formal regulative institution, we focus on the carbon price that affects the structure of rewards and costs, nudging the economy to restructure. We test this institution in the IAM and EEO models that are designed for exploring either macro-economic or sectoral effects of such changes in economic incentives. Given the strengths of ABMs, we use the model to test the effects of two informal institutions: normative (here, a descriptive social norm, i.e., a perception of what behaviors are typically performed by others in one’s network) and socio-cognitive (here, cognition around decision-making involving attitudes toward sustainable behavior and learning). We mostly define all three types of institutions as exogeneous and static, to expose the dominant traditions regarding institutions in sustainability modeling. Given how important endogenously evolving institutions become for sustainability transitions (10, 23), we explicitly embed dynamic endogenously changing social norms in the ABM to highlight the state-of-the-art in representing institutions. With each of the IAM, EEO, and ABM models, we run two sets of experiments: “Reference Institutions” and “Heterogeneous Institutions.” For the ABM, each of these two sets of experiments was performed with 100 Monte Carlo runs. Additionally, a third set of experiments was run with ABM to compare static and dynamic social norms (*SI Appendix*).

**Fig. 3.**
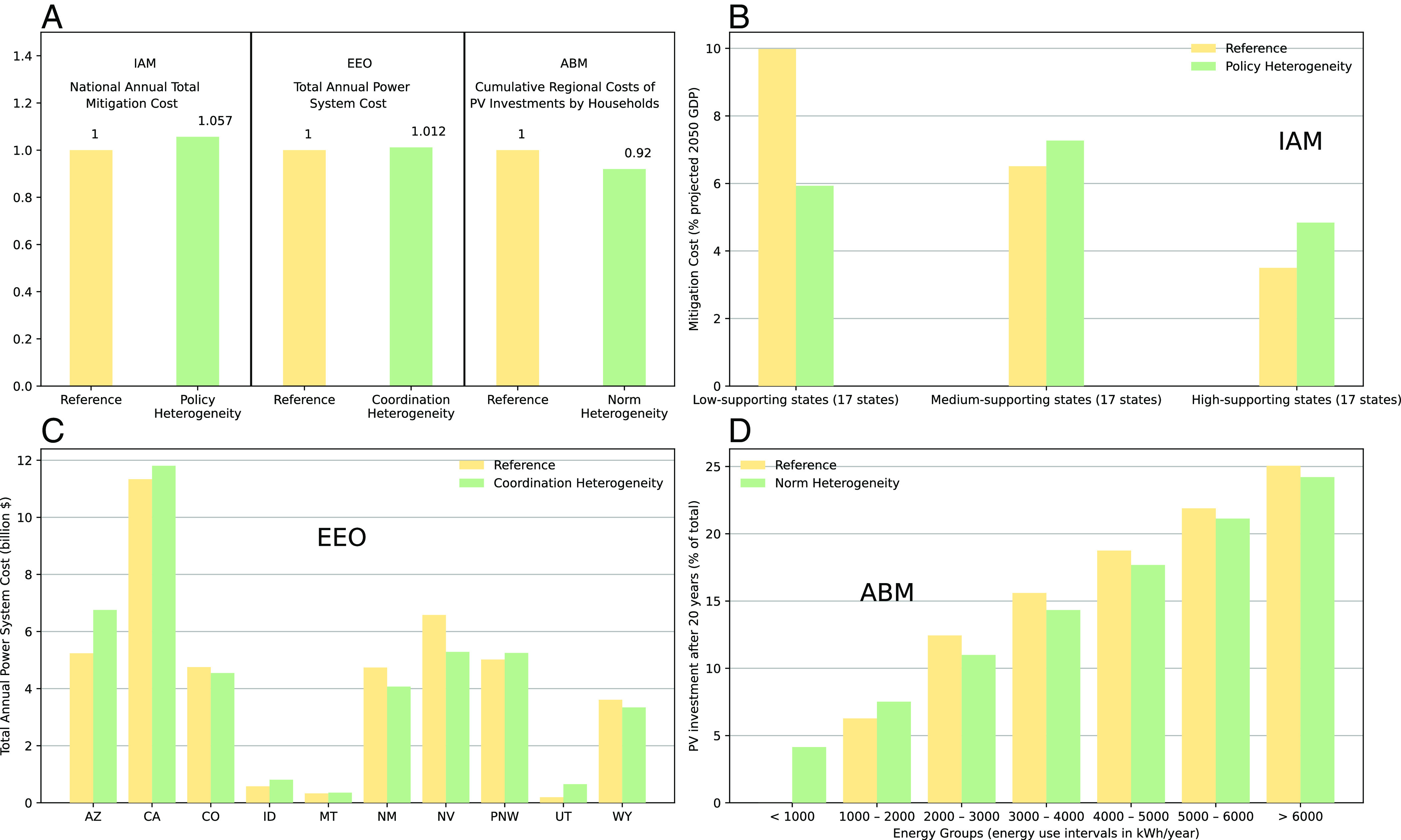
Aggregate (*A*) and distributional impacts (*B*–*D*) of explicit heterogeneous institutions. Distributional impacts are larger than in the aggregate and may differ directionally in three sustainability models.

Fig. 3*A* shows one key outcome of interest: economic costs of the mitigation technology system. Both IAM and EEO show cost increases to meet given policy targets when considering the selected institutions, at levels of a few percent. The ABM, by considering informal socio-cognitive and normative institutions that affect household solar deployment patterns, shows overall lower investment compared to the case without institutions, thus reducing the level of mitigation. As compared to aggregate impacts, distributional impacts of the scenarios indicate much larger changes in overall composition and incidence (Fig. 3 *B*–*D*). In particular, for IAM and EEO, subnational regions (in this case, U.S. states) that have lower policy support and/or higher institutional barriers to regional integration show lower total deployment (and hence investment or mitigation costs) when considering institutional heterogeneity. The ABM, including attitudes and social norms associated with behavior like investing in solar PV in addition to financial considerations, reveals that for the four middle classes of households in terms of reported energy use, these informal institutions primarily serve as barriers that reduce otherwise economically efficient PV adoption. In contrast, the two very low energy consumer classes do not adopt solar or adopt less in the absence of these informal institutions compared to strict financial considerations. Therefore, attitudes and social norms tend to reduce variations in outcomes among different households. Explicitly accounting for the influence of endogenously changing institutions, such as social norms, by contrast, can increase adoption relative to the reference case. Specific results for each set of simulation experiments are described next.

### IAM Experiments: Costs of Subnational Climate Action.

Traditional assessment of decarbonization cost with IAMs assumes idealized policy action such as a nationally uniform carbon price. However, real-world policy is highly heterogeneous and varies substantially across subnational units according to levels of political support and administrative capacities. Using a process-based state-level IAM for the United States (GCAM-USA), we incorporate heterogeneous policy action at the subnational scale and implement it as state-varying carbon prices to proxy for future policy stringency; these variations are based on current public support levels using public opinion surveys (54). Compared to the Reference Institutions case where we deploy a uniform carbon price, in our Heterogeneous Institutions case, we implement this institutional variation through different levels of state-level carbon prices (changing by more than a factor of 3). More information about the model experiments and results can be found in ref. 55.

We find that the nationwide mitigation cost is higher with state-varying policy efforts, though the magnitude of cost escalation is less than 10% for a wide range of national decarbonization targets. For instance, to achieve 80% decarbonization by 2050 relative to 2005 (Fig. 3*A*), the national mitigation cost for the Heterogeneous Institutions case (i.e., state-varying carbon prices) is only 5.7% higher than the Reference Institutions case (i.e., nationally uniform carbon price). Yet, this small increase in national cost comes with a different subnational distribution (Fig. 3*B*): moving from nationally uniform to state-varying action, the economic cost of mitigation drops in the low-supporting states (i.e. the bottom 1/3 states with lower public support level) by up to half; the medium- and high-supporting states (i.e., the middle ⅓ and upper ⅓ of states) take up most of that slack.

Importantly, our sensitivity analyses identify two key factors to keep the costs of state-varying climate action low: modest efforts even by the lowest supporting states and inter-state trade of energy products (see quantitative discussions in ref. 55). If energy markets are tightly coupled across states when all the states are committed to at least a modest floor level of efforts, the high-supporting states can tap into lower-cost mitigation options in the other states through the trade of electricity and bioliquids. This core insight from our IAM experiment highlights the importance of our next experiment using EEO to carefully examine electricity market institutions and trade barriers.

### EEO Experiments: Institutional Frictions and Renewable Energy Deployment.

Electric power systems planning models optimize investment and production cost subject to constraints related to electricity supply and delivery. In the most common configuration, a single decision-maker (i.e., central planner) seeks to minimize total system costs—an outcome that is mathematically equivalent to perfectly competitive energy markets ignoring non-linearities (56). However, many countries and subnational regions lack standard designs and centralized markets or have other barriers to electricity trade. Using the case of the western United States, we optimize a zero-carbon power sector in 2050 under different institutional assumptions. Compared to the Reference Institutions case with the single central planner assumption, the Heterogeneous Institutions case applies a set of regulatory and market institutions present in the western United States that originate from the lack of a single power system coordinator. Specifically, these institutions generate inflexibilities in the trading of power and sharing of resources to meet peak electricity demands without rationing.

We find that considering this subset of real-world institutions raises the cost of meeting the 2050 zero-carbon target by a few percent (Fig. 3*A*). The small overall change reflects the selected institutions modeled and hides more significant distributional changes in system costs driven by deployment of renewable energy and complementary infrastructure such as storage and transmission (Fig. 3*C*). For example, with Heterogeneous Institutions giving preference to local resources and limiting the trade of electricity across large distances, the state of Arizona deploys 167% more solar and 628% more storage capacity compared to Reference Institutions (*SI Appendix*, Fig. S2.1). The minor cost penalties but significant heterogeneous incidence are similar to findings from the more aggregate IAM. The EEO still examines cost minimization, here subject to various constraints, and does not modify assumptions about informal institutions that might alter financial considerations for deploying renewable energy. This is explored next with the ABM.

### ABM Experiments: Informal Institutions Can Hinder or Facilitate Energy Transitions.

**Fig. 4.**
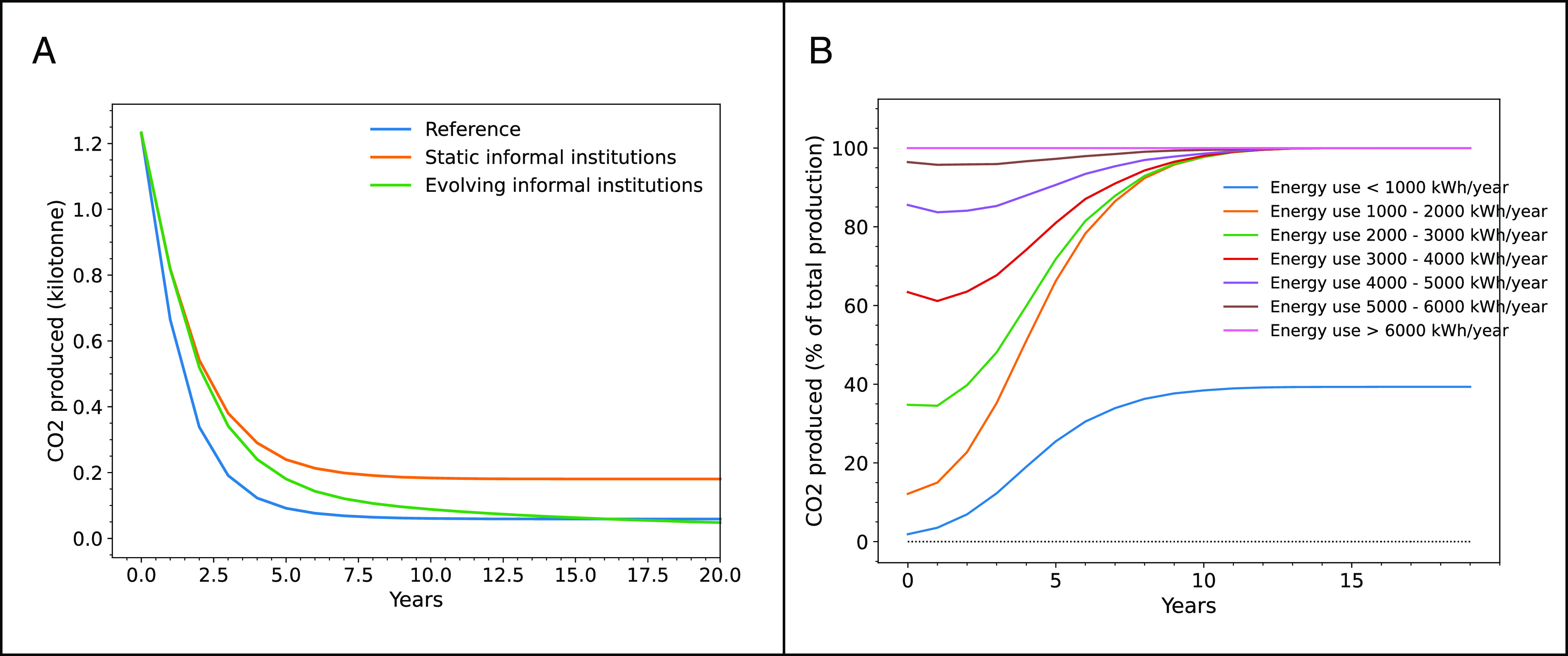
Evolution of emission reduction trends over time, (*A*) aggregated at the regional level under various assumptions about institutions; (*B*) distributed across households with various energy consumption, the default case without institutions, i.e. with solar panel adoption by households driven only by economic considerations.

Adoption of green energy technologies is crucial to speeding energy transitions. Yet, in practice, households’ adoption of green technologies lags behind the optimal level, revealing the “energy efficiency gap” of household non-adoption of energy-saving measures (57). Using an ABM parameterized with the survey data from the Netherlands (58), we study regional trajectories of solar panel adoption among diverse households, changes in regional CO2 emissions and in private investment costs, in the presence (or not) of socio-cognitive and normative institutions. For the Reference Institutions case, we assume that households make decisions on whether to invest in solar PVs based only on financial considerations (*Materials and Methods*). In the Heterogeneous Institutions case, we incorporate informal institutions in terms of the influence of diverse individual attitudes and social norms toward installing PV, in addition to the traditional financial motives. Heterogeneous attitudes and social norms are derived from empirical distributions elicited via households’ survey (58). Furthermore, in another set of experiments, we allow for the endogenous evolution of social norms influencing opinion dynamics among households to explore whether and how evolution of this informal institution can bridge the demand-driven energy-efficiency gap. We perform 100 Monte Carlo runs with the ABM for each of the experiments (Reference; with static informal institutions to which we refer as “Norm Heterogeneity” case; and with evolving informal institutions). Figs. 3 and 4 present the averages across these runs.

Our results indicate that in the presence of heterogeneous normative and socio-cognitive institutions, the diffusion of solar panels among households in the region is reduced (Fig. 3 *A* and *D*), and that CO2 savings due to installed panels are nearly 10% lower compared to the Reference Institutions case (Fig. 4*A*). Hence, the latter serve as barriers to the adoption of technologies compared to what is economically efficient. Notably, the effects of these institutions are heterogeneous, with low-income households increasing their adoption. However, when incorporating endogenously changing institutions by having households exchange opinions about their attitudes to solar panels—mimicking social norms evolving over time—the diffusion of PVs accelerates. Depending on the speed and strengths of opinions (i.e. green uncertainty intervals in Fig. 4*A*, and sensitivity analysis in *SI Appendix*, Fig. S3.2), the uptake of residential solar can overshoot the economically efficient level of PV adoption by households, hence accelerating energy transitions in line with evolving social norms.

Notably, as ABM models choices of individuals heterogeneous in incomes, attitudes, and energy use here parameterized from empirical survey distributions, we can also observe which groups pioneer in their contributions to the energy transition (Fig. 4(*b*)). For example, our results suggest that households with high energy usage (above 2000 KW annually), would be the first to switch to solar; while for households with smaller energy use it does not appear attractive in the absence of institutional influences.

## Discussion

Computational models commonly make implicit assumptions regarding institutions of sustainability-relevant sectors and ignore the heterogeneity of political systems, attitudes, norms, and interest groups shaping sustainability outcomes. By abstracting geographic and sectoral differences in institutional make-up, these models tend to overestimate likely policy homogeneity and underestimate implementation and integration difficulties. Real-world institutional heterogeneity includes formal institutions governing markets such as specific market designs consequential for technology deployment, political institutions that shape the relative power of incumbent and emerging coalitions of actors, and regulatory institutions that determine the terms of trade and competition in commodities such as energy. Heterogeneous informal institutions deriving from individual and group differences in norms, values, beliefs, and ways of learning can also challenge the generalizability of sustainability analyses.

Our findings reveal that even incorporating only one of many institutions in computational models delivers measurable effects on sustainability outcomes, such as emission reductions (8-11%) and costs (~6% higher nationwide cost). Furthermore, the incorporation of heterogeneous institutions can have directionally different effects depending on the model choice and level of disaggregation. Importantly, the reported effects are constrained by the rigidity of contemporary models that are typically not designed to capture a variety of institutions, let alone permit them to evolve endogenously. Therefore, these results should be viewed as demonstration of the minimum measurable effects of incorporating (mainly) static institutions, not an upper bound on the implications of heterogeneous institutions for sustainability. Testing the effects of various institutions, individually and jointly, is an important direction of future work. Furthermore, inclusion of interacting formal and informal institutions that could amplify one another is important, as some preliminary work indicates that they have synergistic effects (43).

Findings from two equilibrium/optimization models (IAM, EEO) inherit a consequence of their cost-minimizing nature: They are effective at optimizing around constraints (in this case, institutional constraints), but these results still depend on an overarching implicit institutional environment favorable to optimization. Distributional effects, which can have greater impacts on political feasibility and institutional development, are much larger than aggregate impacts. Optimization models not attuned to these impacts may inadvertently accentuate the inequity of costs and benefits. Alternative approaches changing the objective or using multiple criteria may be used in conjunction to address this gap (34).

Findings from the ABM experiments reveal a perspective on the importance of institutions in the presence of feedbacks. When considering endogenous institutional change (i.e., evolution of social norms), these adaptive processes have the potential to negate some of the penalties associated with institutional considerations, in some cases even leading to “over-compliance.” Notably, the ABM presented here incorporated only a very narrow set of institutional factors affecting behavior. A multitude of those that do matter for demand-side solutions (59) could not be incorporated here due to the lack of data. Those ABMs that do incorporate these behaviors find that differences imposed by normative and socio-cognitive institutional aspects could diminish emission savings won by the residential solar adoption by 63 to 80% compared to the case where purely economic considerations affect households choices (44), and that modeled patterns reproduce reality (60).

Through incorporation of explicit institutional detail in three commonly used sustainability models, we also aim to shed light on the pathways to more expansive institutional analysis. In Table 1, we highlight the key insights from each of these models as well as limitations that indicate the opportunities for additional analysis and modeling approaches. These model advantages and limitations represent a broader scope than what is included in model intercomparison projects which compare results of roughly similar models. As we move from macro (IAM) to meso (EEO) to micro (ABM), models can incorporate different types of institutional detail, ranging from population-level differences in policy support and institutional capacity, and market and formal institutional differences in energy sector operations, to household heterogeneity and informal institutions shaping green energy technology adoption decisions. We observe that more granular models can be used to address limitations in aggregate models—e.g., EEOs representing trading institutions not present in IAMs, and ABMs representing household-level informal institutions not present in EEOs—but this approach may run into computational limitations (limiting, e.g., geographic scope), data gaps, or both.

**Table 1.** Insights and limitations of incorporating institutions across the three types of sustainability models

	IAM	EEO	ABM
Insights	Decarbonizing via bottom-up climate policy can be cost-effective, provided flexible inter-state electricity trade	Institutional coordination barriers in the power sector can shift infrastructure deployment patterns and mitigation incidence	Accounting for household-level normative and socio-cognitive institutions helps explain suboptimal residential solar uptake
Limitations and additional modeling accounting for institutions	Trade and market institutions–such as balancing, state-state political interactions and policy coordination–not well captured	Household decisions (e.g., electrification, distributed energy adoption, responsive demand) are treated exogenously	Converting the capabilities of conceptual ABMs into modeling formal (markets, policy) and informal (norms, attitudes, socio-cognitive processes around learning) institutions relies on massive empirical microdata

We identify several potential opportunities within this set of three models for expanded research questions. To date, computational sustainability models have mainly been used to examine a simplified set of formal institutions, typically in the form of exogenous scenarios: policy targets, constraints, costs, and price-based incentive policies (taxes and subsidies). Yet, even without changing the current model structures, models can be used to tackle other institutional questions that directly touch on the political economy of sustainability transitions. In Table 2, we list an expanded set of questions that these models can study. These include the exploration of incumbent institutions such as carbon lock-in, legacy market impacts, and the effects of non-economic incentives on adoption. In addition, in larger systems, institutional and market frictions become increasingly important, such as cross-sector integration, barriers between market zones, and the interaction of competing norms.

**Table 2.** Example institution-related research topics and questions that can be addressed by expanded sustainability modeling

	IAM	EEO	ABM
**Traditional Efforts:** Policy target constraints and costs	How do different global climate cooperation frameworks affect the optimal mitigation policy?	What is the least-cost electricity system that achieves a 90% GHG reduction?	How do heterogeneous boundedly rational households respond to institutional/policy drivers?
**Emerging Research:** Incumbent institutions	How might carbon lock-in and stranded fossil assets limit changes to the emissions trajectory?	What is the impact of inflexible trading and contract arrangements on renewable energy integration?	What are the impacts of market-based vs. information policies on diffusion of low-carbon technologies?
Institutional and market frictions	How might risk-aversion in the finance sector hamper low-carbon energy transitions?	How do coordination barriers between adjacent electricity jurisdictions affect planning and operations?	To what extent could social norms inhibit or facilitate low-carbon energy transitions?
**Next Frontier:** Endogenously evolving institutions	How would institutional quality affect the credibility of countries’ climate commitments, hence the future of international climate negotiation’? How would the changing geopolitical tensions and trade environment influence the technology supply chain and deployment over time? How would domestic economic and political interests influence technology and financial decisions within and across countries?	At which stage in the low-carbon energy transition might we see enhanced pressures for governments and utilities to coordinate? How might market design adaptations to flexibility needs of low-carbon energy affect trajectories of deployment? How does the balance of organized interest groups (e.g., utility vs. distributed generation) co-evolve in response to policy and deployment?	What is the role of dynamic institutions - restructuring markets, changing policies, shifting social norms - in enabling socio-economic tipping points in sustainability transitions? How do new policies, such as ambitious sustainability targets or transformational turns in policy, emerge at the system level as a consequence of changes in individual behavior and/or preferences?

### Toward a Research Agenda for Including Social Institutions in Nature–Society Models.

Models employed in sustainability research do already account for some institutions, though they may enter the modeling efforts implicitly. Increasingly, we note the inclusion of formal market and regulatory institutions explicitly in models of nature–society systems. Where incorporated, these institutions typically remain static over the course of a simulation, thus limiting their effectiveness for understanding transitions. As various sustainability challenges demand more bold and transformative actions, which go beyond marginal market changes or incremental behavioral changes, understanding and capturing the dynamics of various institutions—formal and informal—becomes crucial.

A central part of the modeling challenge is to identify which institutional factors can be represented quantitatively in models and, given the strength of empirical evidence and theoretical foundations, what is the suitable model choice and strategy. For instance, when only qualitative evidence is available or competing theories still exist, quantitative representation of these factors is unlikely to yield solid quantitative conclusions. These factors are thus better treated outside of the numerical models; they can be represented as scenario narratives as a way to generate qualitative insights into their importance, or be used to create relevant indicators for feasibility assessment (27, 61, 62). When quantitative evidence is available, it can be used to constrain model assumptions and parameter values to align better with socio-political realities (63, 64). When strong empirical relationships are available (for example, learning rates and varying demand elasticities across income groups), incorporating these relationships in a reduced form can be a fruitful endeavor for advancing models. In short, finding the right modeling approach requires first assessing the strength of social science evidence and then choosing the modeling strategy based on the weaknesses and strengths of different model types and methods. For instance, global IAMs are perhaps least useful in informing nuanced policies at decision-relevant scales because they lack the capability to represent the huge uncertainties and heterogeneity in subnational politics, interests, and decision-making processes (65). In contrast, ABMs, EEOs, or subnational IAMs provide a more promising framework to incorporate relevant institutional factors that reflect sector- and region-specific considerations.

Recognizing these epistemological challenges, below we outline a number of directions where the interdisciplinary community concerned with sustainability, and of nature–society interactions in general, could focus to improve upon modeling institutions.

1. **Collecting empirical evidence on (evolving) institutions:** Embedding institutional considerations into models requires advancements not only in models but also in our understanding of social systems. The success of this integration requires close collaboration between modelers and social scientists to identify how the models could incorporate institutions in tractable ways and be anchored in the relevant social sciences (18, 25, 26). Changes in social institutions may occur infrequently or take decades or even centuries to occur, hence limiting systematic empirical evidence. Over very long time horizons, there is some progress using data collected by historians and archeologists to capture changing institutions for modeling purposes (66, 67). At the same time, rule-making affecting market and regulatory institutions occurs on a near-continuous basis, generating in some cases a wealth of untapped data (68). For multi-sector models such as IAMs, a full upgrade would also require empirical evidence and model representations of how actors behave differently across a wide range of sectors.
2. **Systematizing the theoretical base for various institutions:** Furthermore, research on the theory of evolving institutions is scattered across a range of social science disciplines: market institutions by various branches of economics, policy change by public administration scholars, political institutions by political scientists, normative and social–cognitive institutions by sociology and psychology scholars, as well as sustainability scholars in general across all of these. The right choice in terms of the type of institutions—formal or informal—and their representation—static or endogenously changing—depends on the nature of the theoretical knowledge and evidence these different social sciences offer. Furthermore, these, typically qualitative, theories of institutional change must be formalized in a computer code, upon which most sustainability models rely.
3. **Beyond implicit assumptions on institutions in (hybrid) models:** The easiest and currently most widely used path to integrate qualitative insights into institutions into models is by translating them into narratives of “what-if” scenarios (69), exploratory “conceptions of reality” (7), or as metrics to assess the scenarios (61). As more data become available, quantitative insights are increasingly used to constrain model assumptions to better reflect the socio-political considerations that represent regulatory and market institutions (29, 55, 70). Nevertheless, EEOs of realistic energy systems typically require linearity to maintain computational tractability, limiting the complexity of institutional forms. Hybrid models offer the means for sustainability scientists to capture interactions between formal and informal institutions, such as market institutions reinforcing the effects of socio-cognitive and normative institutions (71). Among the model combinations presented here, agent-based IAMs have simulated heterogeneous agents (e.g., individual goals, bounded-rationality, imperfect foresight) in macro-energy systems (30, 45). Hybrid EEO/ABM models have examined short-term (e.g., market bidding) as well as long-term (e.g., planning and transition) decisions by energy firms, for example, by embedding a detailed optimization in a larger ABM considering heterogeneous firm characteristics and preferences (72, 73). These hybrid models provide an avenue to improve the institutional and behavioral realism of the modeled decisions, while also creating new analytical challenges with respect to computational complexity and tractability.
4. **Institution-making processes relevant for sustainability science:** Modeling approaches laid out here can also provide insight into the policy-making process. As initial priorities for exploration, we propose investigation of the following institutional processes: 1) policy target ratcheting up (e.g., processes of strengthening targets over time); 2) policy change in response to interest group reorganization and the formation of new coalitions of power; 3) enhanced policy coordination in response to frictions with distinct policy systems; 4) policy diffusion; 5) structural changes in markets leading to the emergence of new actors and systemic shits in prices and investments incentives; and 6) interactions between changing formal and informal institutions, like shifting social norms of individuals (also consumers-voters) impacting markets and triggering a policy shift.
5. **Endogenizing changes in institutions in sustainability models:** Many sustainability transitions require changes in various institutions, both formal and informal. In rare cases where empirically assessed relationships are ripe for inclusion in models, critical processes that drive changes in institutions can be endogenized in a model. Where such relationships and data on the evolution of social institutions over time is unavailable—or the shift has not happened yet—one could still perform exploratory modeling based on the dominant social theories of institutional change, like theories of policy process (74), (qualitative) narratives of experts or stakeholders about the anticipated changes in institutions. In either case, we foresee two options for integrating dynamic institutions in sustainability models. A pathway that would not require major restructuring of the traditional models is to employ a reduced-form representation of dynamic institutions, such as learning rates and relevant evidence to model endogenous technological change (23, 75). Alternatively, one may pursue modeling the processes of institutional change endogenously, such as changing policies and regulations due to falling prices, or restructuring markets and the emergence of new policies as attitudes in the society shift. Although this is computationally complex, such models based on empirical data are already in use (66, 76), though mainly for incremental changes in institutions. Endogenous descriptions are generally more sensitive to both parametric (lack of data) and structural uncertainty (specific equations to capture endogenous institutional change), which has systemic impacts on model validation and insights (77, 78). Addressing the latter requires re-coding the structure of standard models, which might take years for large models, appear contradictory to their foundational principles, meet resistance in the modeling community, and lead to trade-offs in the realism of the techno-economic representations. Yet, given that models with omitted or static institutions could underestimate the costs and effectiveness of policies, it becomes increasingly vital to pay attention to how social institutions are represented in formal sustainability models.

Before diving into the massive effort of endogenizing institutions, one could assess the possible scale of the impact, e.g., the size of the potential effects on key outcomes. One should use such tests on existing models with care, as they inherit the assumptions of implicit institutions, usually with little flexibility to assess the influence of alternative assumptions. Modeling approaches should be guided by the strength of the empirical evidence and our understanding from other epistemological traditions on the future evolution of social systems.

Nevertheless, with the climate and biodiversity crises accelerating, we cannot expect the institutions working in the past to continue operating effectively. This means that modeling spanning long-term horizons would need to involve adaptive human actions and institutional responses, at minimum, explicitly testing implications of novel institutional arrangements and possibly estimating thresholds that drive them. In addition, as socio-economic, political, and environmental systems are interconnected, cascading effects from one system to another can lead to local or global collapses. Given this evolutionary nature of complex adaptive nature–society systems, policy scholars, for example, increasingly collect evidence on “ecology of games” and cycles of eroding and reviving policy institutions (79, 80).

The modeling experiments performed in this paper demonstrate some preliminary efforts in the context of climate mitigation. The next key challenge is to identify promising modeling strategies that are worthwhile to invest time and test out. There are cases where endogenizing institutions are likely to yield high returns. For instance, incorporating a wide range of formal and informal institutions in a stylized model coupling climate and social systems (e.g., DICE) has produced high-level insights for potentially important feedback loops (23). A crucial next step is to represent relevant feedback loops in more technology-rich, decision-relevant models, like the three types of models presented in this paper, to guide concrete decisions made by the market, policy, and individual actors. In other cases, instead of making each model increasingly complex, one could pursue coupling methods to link distinct models to reflect processes and institutions that are relevant at different scales and granularity. For instance, our results indicate that institutions affect social welfare and distributional outcomes in each of the models—and point to the need for careful interaction between insights at different scales.

## Materials and Methods

The models, simulation settings for relevant institutions, and sets of experiments performed in the paper are described below and summarized in Table 3.

**Table 3.** Design of the simulation experiments to explore the representation of different institutions in the IAM, EEO, and ABM

	Model		
Experiment settings	IAM	EEO	ABM
Experiment 1: “Reference Institutions”	Decision-making by a central planner that requests uniform mitigation actions across states Type of institution: Regulatory Nationally-uniform climate policy stringency modeled as carbon pricing Type of institution: Market Unconstrained transmission within each grid region Unconstrained transport of bioliquids across states	Decision-making by a single central planner with perfect coordination. Type of institution: Market Hurdle rate = 0 Operating reserves = Single region-wide requirement Type of institution: Regulatory Resource adequacy = Single region-wide requirement	Decision-making as a rational process based on estimating Net Present Value. Type of institution: Normative *SocialNorm* = 0 Type of institution: Socio-Cognitive *Attitude* = 0
Experiment 2: “Heterogeneous Institutions”	Policy stringency: state-varying carbon prices based on current public support rates	Hurdle rate: penalties for electricity flows between load zones that are not part of a single coordinating market Operating reserves: separate reserve requirements for each load zone Resource adequacy: separate requirements to meet peak load for each load zone	Decision-making as a behavioral process. *SocialNorm* = sum of socially-connected households having PVs installed, weighted according to relative importance reported in survey data. *Attitude* = normal distribution parameterized using the survey data, weighted according to relative importance reported in survey data.
Experiment 3: “Dynamic Institutions”	n.a	n.a.	*Attitude* parameter for each individual agent evolves over time following opinion dynamics

### Integrated assessment modeling (IAM).

We use the GCAM-USA model, which is a state-level model embedded in a global multi-sector, multi-regional model (75, 81). The model simulates interactions between five broad sectors: socioeconomics, energy, land, water, and climate. GCAM-USA divides the energy and economic systems of the United States into 50 states and Washington DC, with state-level representation of socio-economics, energy transformation (power generation and refining), carbon storage, renewable resources (wind and solar), electricity markets (with the representation of regional electricity grids), and consumer end-use energy demands (in buildings, transportation, and industrial sectors).

We perform two sets of experiments: 1) Reference Institutions: least-cost mitigation pathway with a nationally uniform carbon price to achieve economy-wide deep decarbonization by mid-century; 2) Heterogeneous Institutions: state-varying climate policy stringency, represented as heterogeneous carbon prices, based on the public support. The details for these experiments are represented in ref. 55. The two scenarios selected as examples in this paper are the two main scenarios achieving 80% nationwide decarbonization by 2050 compared to 2005 levels, i.e., “80% uniform” and “80% heterogenous” in ref. 55. The outputs from GCAM-USA not only include CO2 emissions and mitigation costs that are key variables for this paper; it also includes sectoral production, technology choices, and trade of energy products across states, which serve as the basis to understand the emissions and costs outputs.

### Engineering–economic optimization (EEO).

We develop an engineering–economic optimization model for eleven states of the western United States that solves for power system capacity expansion and operations. Capacity expansion models with hourly operational detail are used to evaluate important techno-economic trade-offs among generation, transmission, and storage technologies in order to meet demand, particularly important when considering new intermittent renewable energy sources (82). The model evaluates optimal capacities of various resources to meet state-level demands and power system operations (power flow, generator dispatch, storage charging and discharging, operating reserves, and thermal unit operations) in 2050 for a zero-carbon power sector with 100% clean electricity sources. It is a mixed-integer linear programming model whose objective function minimizes system cost, including investment and fixed costs of resources as well as variable costs of generation and unmet demand.

In our Reference Institutions case, we take the model as traditionally structured, which assumes perfect coordination among the different load zones and sharing of resources to meet reserves and peak demand resource adequacy. The lack of a western-wide regional transmission operator and the presence of transaction costs for trading electricity across zones presents institutional hurdles to achieving this least cost outcome (83). The implementation of institutional heterogeneity in the Heterogeneous Institutions case consists of a) “hurdle rate” penalties for electricity flows between load zones that are not part of a single market, b) separate operating reserve requirements for each load zone representing the lack of coordination, and c) separate resource adequacy requirements (considered with fixed import/export assumptions resulting from resource adequacy programs and long-term power purchase agreements) such that each zone must have sufficient resources to meet peak demand within its borders. Sources of institutional information for these parameterizations come from government planning documents and historical flows.

### Agent-based modeling (ABM).

To illustrate the effect of modeling (cognitive) institutions on an individual (household) level, an agent-based model (ABM) on the adoption of solar rooftops was designed. Specifically, the ABM focuses on the installation of PV panels by households in the Netherlands. The agents in the model are heterogeneous in income as well as in yearly energy consumption. The simulations cover a timespan of 20 y, where a single timestep equals one year. Income and energy consumption are independent, and the distributions of these parameters are taken from previously collected survey data on household solar panel adoption in the Netherlands (N = 735) (58), and are hence heterogeneous across household agents.

In the model, three types of decision-making processes are implemented. First, the model is run with all rational agents, who base their decision to install PV panels only on financial considerations; we call this scenario Reference Institutions. Specifically, they compare the utility of installing PV panels to the utility of not taking action by computing the net present value of both options. If the utility of installing PV panels is higher than the utility of not doing so, and the household has enough savings to pay for the costs of installing PV panels, the panels are installed. It is assumed that every household installs enough PV panels to entirely fulfill their energy consumption needs. Second, the decision-making process is extended with a number of behavioral factors that affect individual choices, and which correspond to socio-cognitive and normative informal institutions. This decision-making process follows the Theory of Planned Behavior (TPB) (84), according to which households base their decisions on personal behavioral control (PBC), which is represented by financial considerations using the same method as for the rational agents, as well as on their attitude toward installing PV panels (that represent cognitive process), and on social norms, which concern the actions and/or opinions of others, and hence serves as a proxy of normative institutions affecting households choices. The agents are heterogeneous in attitude values (*Attitude*), which are parameterized using the survey data mentioned above. The social norm (*SocialNorm*) is implemented as the actions of households’ connections in a social network; we use the survey data also to parameterize social networks of households. The decision-making process is again formed by comparing the utility of installing PV panels to the utility of not installing PV panels, but here both utilities depend on attitude and social norms as well as on the NPV. The weights of each of these function components are determined using the survey data mentioned above, from which several decision-making groups could be distinguished, which differ in their motivation to install PV panels. For example, some groups indicate they are only motivated by themselves, which translates to only giving weight to their own attitude, while others might indicate that they are motivated by financial reasons as well as by their social contacts, which translates to giving weight to PBC as well as to social norms.

Finally, we add opinion dynamics to the model, in which the attitude values of the agents can vary over time under the influence of the attitudes of others in their social network. The opinion dynamics are implemented such that every household can give different weight to each of its connections, and these weights do not change over time. Households’ attitudes are updated every timestep according to a certain influence rate parameter (μ) that determines the rate at which all households are influenced by the aggregated attitude of their social connections. This setup avoids guaranteed convergence of the agents’ attitudes, which does not seem to apply in this context. Since there is no data available on how attitudes toward renewable energy adoption change when people interact, the influence rate is varied around 0.1, as specified in ref. 85 and *SI Appendix*, Table S3.2.

To illustrate the effects of modeling cognitive institutions, the results of the ABM are compared for rational and behavioral agents as well as behavioral agents with opinion dynamics. Simulations are compared in terms of cumulative (monetary) investment in PV panels, as well as on the annual amount of CO2 produced in the model. For every simulation, the model is run with 1,000 agents, a timespan of 20 y; we report results averaged over 100 Monte Carlo runs. Results are evaluated as aggregated outputs for all agents and distributed over different energy consumption groups.

## Supplementary Material

Appendix 01 (PDF)

## Data Availability

The IAM datasets generated during and analyzed in the current study are available from a public repository (86). IAM models are available for download for GCAM (87) and GCAM-USA (55). The EEO datasets and model generated for the current study are available from a public repository (88). The ABM model and results are available from a public repository (89). Figures produced for the paper can be reproduced from the repository (88).

## Footnotes

^*^Other sustainability models that incorporate human actions include social network modeling and socio-ecological systems modeling (such as system dynamics) (7).

^†^While we test only 1 to 2 examples of institutions from each of the three classes—regulative, normative, and socio-cognitive institutions (19)—future work could focus on systematically testing all possible institutions (e.g., 25 to 30 examples of institutions for energy transitions in ref. 47). This said, we acknowledge that there are many alternative classifications of institutions conceptualized by various disciplines involved in sustainability research, and that for another application a different set of institutions might need to be explored.
