# Supplementary material for: Simulating institutional heterogeneity in sustainability science
Source: Proc Natl Acad Sci U S A. 2024 Feb 15;121(8):e2215674121. doi: 10.1073/pnas.2215674121 (PMC10895347; doi:10.1073/pnas.2215674121)
Supplement: Supplementary file 1 — Appendix 01 (PDF) [file pnas.2215674121.sapp.pdf]

PNAS

Supplementary Information for

Simulating institutional heterogeneity in sustainability science

Michael Davidson[1,2]*, Tatiana Filatova[3]*, Wei Peng[4,5]*, Liz Verbeek[3], Fikri Kucuksayacigil[1]

[1] School of Global Policy and Strategy, University of California San Diego, La Jolla, CA 92093

[2] Department of Mechanical and Aerospace Engineering, University of California San Diego, La Jolla, CA 92093

[3] Department of Multi Actor Systems, Faculty of Technology, Policy and Management, Delft University of Technology, 2628 BX Delft, The Netherlands

[4] School of Public and International Affairs, Princeton University, Princeton, NJ 08544

[5] Andlinger Center for Energy and the Environment, Princeton University, Princeton, NJ 08544

* To whom correspondence may be addressed: mrdavidson@ucsd.edu, T.Filatova@tudelft.nl, weipeng@princeton.edu

This PDF file includes:

Supporting text
Figures S.1.1 to S.3.2
Tables S.2.1 to S.3.2
SI References

1. Integrated Assessment Modeling (IAM)

The GCAM is an open-source global integrated assessment model (https://github.com/JGCRI/gcam-core/releases; for more information, see (1) and online documentation http://jgcri.github.io/gcam-doc/toc.html). GCAM represents key interactions across the economic, energy, land and climate systems in 32 geopolitical regions in the world. It is a market equilibrium model that solves for the market prices and quantities of a large number of markets simultaneously. It is dynamic recursive with myopic foresight (that is, the model solution in each model period depends on the conditions in that period or periods before it).

In this study, we use GCAM-USA v5.1, which is a version of GCAM with state-level detail in the United States. Like GCAM, GCAM-USA is an open-source model. Detailed documentation for the GCAM-USA model is available online (http://jgcri.github.io/gcam-doc/gcam-usa.html). Here we summarize key model features that are relevant for this study. The model results for all main and supplementary scenarios are available from a public data repository (2).

GCAM-USA divides the energy and economic systems of the United States into 50 states and Washington DC, with state-level representation of socioeconomics, energy transformation (power generation and refining), carbon storage, renewable resources (wind and solar), electricity markets (with the representation of regional electricity grids) and consumer end-use energy demands (in buildings, transportation and industrial sectors).

Economic growth, population changes and climate changes set the scale for energy demands in buildings, transportation and industrial sectors. These demands are supplied by a variety of fuels and technologies. GCAM-USA includes technological detail in all sectors within the energy system. For example, the model includes about 20 different power generation technologies, about 10 different building service types and building technologies that vary across fuel inputs, and about 40 different transportation technologies that vary across modes, vehicle class, vehicle size and fuel input. The model also tracks details about capital stock vintages in capital intensive sectors such as power and refining along with simple algorithms to retire existing stock based on natural lifetimes and economic conditions. Nested logit structures are used to share out new investments across technologies to meet demands. The logit structures create competition between different fuels and technologies on the basis of relative costs (3). It avoids winner-take-all type of responses and helps capture heterogeneity in unmodelled factors across various technology options.

To represent electricity trade, states are grouped into 15 electricity grid regions to reflect electricity market and planning areas. Within a grid region, we assume unconstrained trade and therefore common electricity prices across these states. Trade between grid regions is calibrated to historical levels

to reflect existing economic conditions as well as implied physical transmission capability. In future modeling periods, trade can change from calibrated levels as relative regional electricity prices change. The model also includes flexibility in the location of energy-intensive industries such as refineries on the basis of the relative costs of production across states based on a non-linear logit equation. For oil refining, our scenarios assume that expansion takes place in the states that currently engage in oil refining and that the relative competitiveness of those states remains constant over time. For newer technologies such as biomass to liquids technologies, we assume that the current distribution of production will remain largely static in the coming years, which, in turn implies that biomass production will largely occur in the same locations as today. Note that the model includes representation of negative emissions technologies (such as bio-energy with carbon capture and sequestration, BECCS) in both electricity and refining sectors.

In addition to a detailed representation of the energy system within the United States, the model includes global representations of the agriculture and land-use systems at the basin scale. These systems are all hard-coupled in code. For example, biomass production is modeled in the agriculture and land-use component of the model that creates a competition for land among various land uses such as biomass, crop production, managed and unmanaged forests and livestock. Biomass produced in the agriculture and land-use component is demanded in the energy system. Likewise, fertilizers produced in the energy system are demanded by crops in the agriculture and land-use system. GCAM-USA maintains representations of 31 geopolitical regions outside of the United States; hence, prices and changes occurring within the United States are consistent with international and global conditions.

1.1 Scenario design

To design mid-century scenarios, we set varying levels of national mitigation effort, targeting the national total GHG emissions in 2050 to be 20%, 40%, 60% and 80% below 2005 levels, respectively. We assume linear GHG mitigation pathways from 2015 to 2050 with 5-year interval. Since GCAM-USA is embedded within the global GCAM model and allows for interactions between the United States and the rest of the world through global markets, to avoid cross-country carbon leakage, we set decarbonization targets for other countries based on (4), which are consistent with the 2°C pathway.

We consider two subnational policy approaches to achieve the national targets. Under the Reference case, the model solves a single MAC (and in turn carbon price) nationally to meet the decarbonization target. The MAC is uniform across states, which is determined by the marginal cost to mitigate the last unit of CO2 emissions nationally. Under the Hybrid and Heterogeneous approaches, we allow for heterogeneous MACs across states. We set the relative ratio of state-level MACs based on the

present-day public support level for climate policy, then let the model solve the whole set of MACs for 51 states. Note that the MACs capture the effects similar to a carbon price. A high MAC encourages the deployment of high-cost CO2 mitigation technologies (such as renewable electricity and BECCS) as well as a reduction in overall fossil energy use. As long as the importing activities can reduce energy production and associated emissions within the state boundary, our approach would not further require importing only low-carbon electricity or goods.

Under the Heterogeneous case, we assume a non-linear relationship between the public support level and MAC. Because the United States is a democracy, we use public opinions to proxy for the heterogeneity in stringency of state policies (Figure S.1.1). Under the Heterogeneous approach, we model the widest heterogeneity in policy stringency (by more than a factor of 3) across all the states (Figure S.1.2).

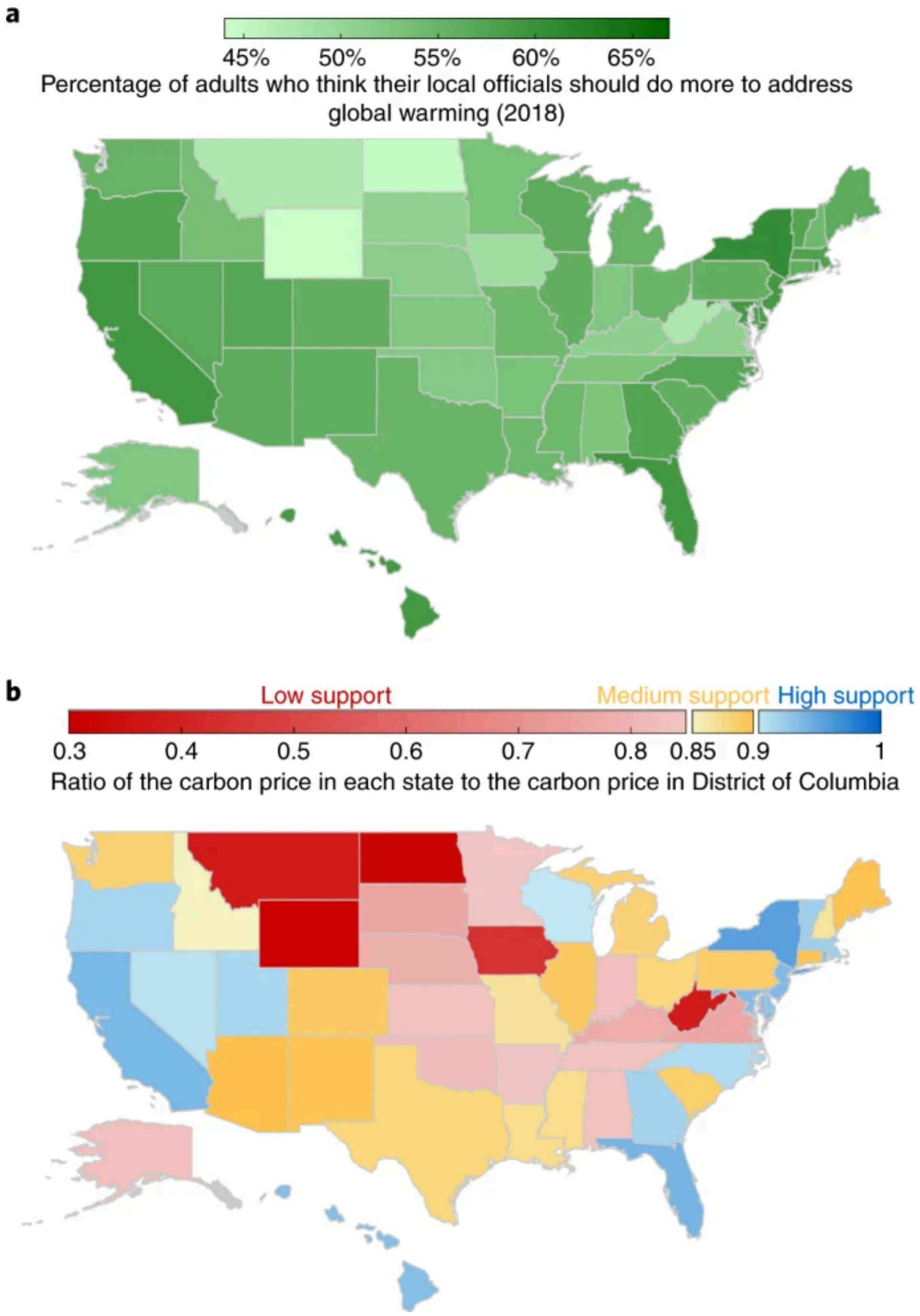

Figure S.1.1. State-level variations in public support for climate policy and its impact on carbon pricing under the Heterogeneous approach. a,) State heterogeneity in public support level for climate policy in 2018. We show the percent of the adult population in each state who think their local officials should do more to address global warming (data from (5)). b) State heterogeneity in carbon prices under the Heterogeneous approach. Here we present the ratio of the carbon price in each state (modeled as the marginal abatement cost of carbon policies) to the carbon price in the District of Columbia, where the support rate is the highest. We group the 50 states and the District of Columbia

into low-, medium- and high-supporting groups (indicated by red, yellow and blue, respectively; each group includes 17 states), based on their current climate policy support levels as shown in a. We assume the state-level carbon price varies non-linearly with public support level and decreases dramatically when the support level drops to below 50% according to the median voter theorem. This assumption leads to much lower carbon prices in states with low support rates.

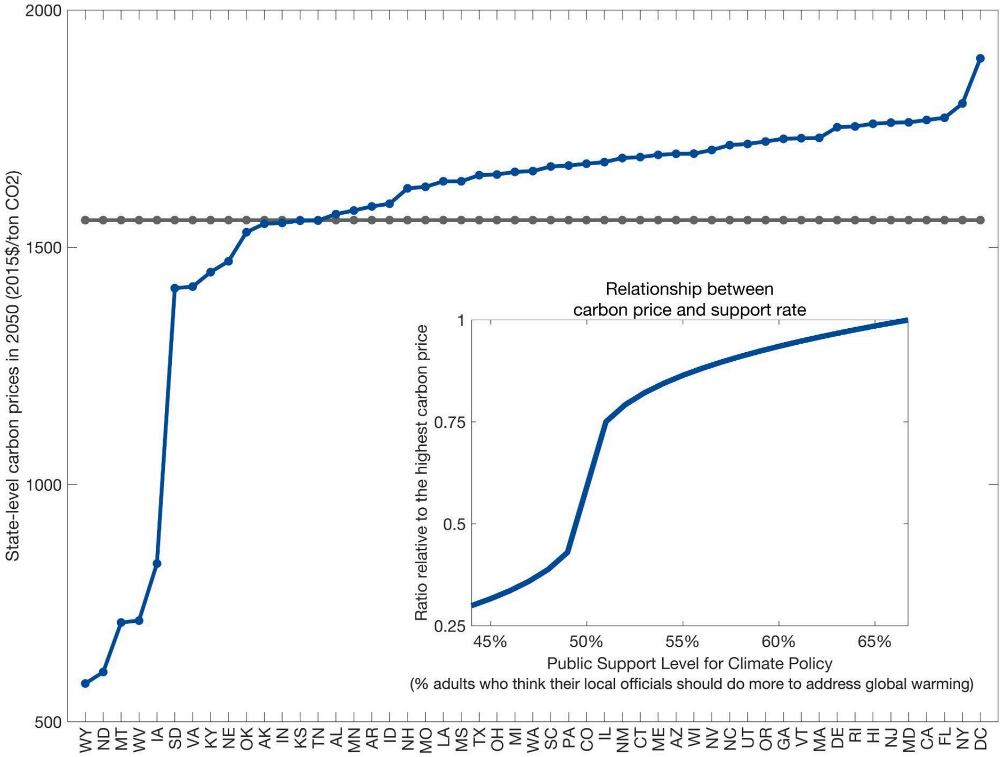

Figure S.1.2. Model-computed state-level carbon prices in 2050 to achieve a national target of 80% decarbonization by 2050 relative to 2005. Blue - *"Heterogeneous Institutions"*; Grey - *"Reference"*. Here, the carbon prices are modeled as the marginal abatement cost of carbon policies in GCAM-USA. This can be interpreted as the implicit carbon price level from adoption of mitigation technologies and behavioral changes. The inset shows different assumptions for how carbon price varies as a function of public support under the *"Heterogeneous Institutions"* approaches, respectively. Based on these assumptions, the GCAM-USA model then computes the whole set of state-level carbon prices to achieve the national decarbonization target, as presented in the main figure.

1.2. Calculation of mitigation costs

To calculate the mitigation cost, we construct state-level MAC curves using the CO2 emissions and MACs from 20% to 95% decarbonization target runs. The model considers state-varying and time-varying MAC curves, driven by the variations in a wide range of factors such as existing

infrastructure, local availability of low-carbon resources and the evolving costs of technologies over time. We then integrate the area under the MAC curves in each state to calculate state-level mitigation cost, and add up the cost to the national level. All monetary values in this paper are presented in 2015 US$.

2. Engineering-economic optimization (EEO)

We build a mixed-integer linear programming capacity expansion model with hourly operational details. It determines optimal capacities of resources and transmission lines for 2050, including expansion and the retirement decisions, and operational decisions for these resources for select weeks over the year. Constraints are elaborated in the next section.

Table S.2.1. Technology classifications in the engineering-economic optimization

Set	Included technologies
CES-eligible	CCS coal, CCS gas, nuclear, biomass, solar, solar thermal with energy storage, wind, reservoir hydro, run-of-river hydro, geothermal
Economic dispatch	Solar, solar thermal with energy storage, wind, run-of-river hydro, geothermal
Non-dispatchable	Solar, wind, run-of-river hydro
Storage	Battery, hydroelectric pumped storage, solar thermal with energy storage
Unit commitment	Biomass, coal, gas, nuclear, other peaker

2.1. Constraints

Bounds on new-build capacity: New build capacities are bounded by potentials which refer to maximum installation amount of geographically constrained resources. These limits are based on land availability and the availability of solar, wind, water, and geothermal heat.

Bounds on generator output: Non-dispatchable units cannot produce more power than weather conditions allow. Unit commitment resources produce power driven by their commitment status.

Ramping constraints: Unit commitment resources can change the dispatch amount from an hour to another hour, but with a limited flexibility. Other resources do not have any bound in alternation of dispatch amount.

Non-served energy: Even though it is an impractical and undesired outcome of a power system, demand may not always be satisfied. We have multiple segments of demand response management, each of which corresponds to a maximum allowed unmet amount and cost.

Storage operations: The energy level of a storage unit is determined by the energy level from the previous period and dispatched and charged energy amount in the current period. We consider round-trip efficiency for discharging and charging operations. The energy level of storage units is capacitated. For reservoir-hydro units, energy balance constraints are slightly modified by replacing the charged amount with the inflow parameter. Reservoir levels should not be below a minimum.

Unit commitment states: We use a three-variable formulation to model thermal units' operations. These variables refer to commitment, start-up, and shut-down status at each hour.

Time limits for thermal units' up and down status: Once thermal units start operating, they must run for at least a specified number of hours. Similarly, they must stay non-operating for at least a specified number of hours once they are shut down.

Bounds on power flow amounts: Flow on transmission lines is bounded by line capacities.

Demand-balance constraints: Demand and supply are balanced for each modeling zone. The sum of dispatched power and net international import (equal to 0 for non-border zones) should equal the sum of demand, power used to charge storage units, and power exported to other regions. We consider non-served demand in this equation.

Reserve contributions from resources: For unit commitment resources, the provided reserve is limited by the remaining capacity of committed resources. The provided reserve cannot exceed the ramping

limit. These constraints have the same interpretation for down reserve, except that dispatched power can be reduced until the minimum power limit. For dispatchable non-unit commitment resources, the provided reserve cannot exceed the weather-dependent limits. For reservoir-hydro units, reserve should be less than the ramping limit. Dispatched power can be reduced until the minimum power boundary to provide a down reserve. Reservoir hydro units cannot dispatch power (or provide up reserve) more than its remaining energy capacity in an hour. For storage resources, the up reserve amount is the sum of reserves provided during charging and discharging. The same applies to down reserves as well. Provided reserves are driven by charging decisions. Providing down reserve while charging means absorbing more power from the network. Provided down reserves can be at the most either power capacity or remaining energy capacity of storage units, considering charging efficiency. Provided up reserve while charging means allocation of charging effort to dispatch power, and thus reserve amount can be charging amount at the most. Provided up reserve while discharging is limited by either power capacity or state of charge, considering discharging efficiency. Provided down reserve while discharging is bounded by discharging amount. Provided total up reserve is limited by power capacity considered with discharge efficiency. For non-dispatchable and run-of-river resources, they can only provide down reserves.

Up/down operating reserve: For coordination heterogeneity, up reserve requirement in each zone for each time period is a certain percentage of demand plus a certain percentage of total dispatch from non-dispatchable renewable resources plus a contingency. We calculate the contingency of a zone as the maximum of the following three parameters: (i) the largest unit size of existing thermal resources in this zone, (ii) the largest capacity of power lines connecting this zone, and (iii) the largest unit size of candidate thermal resources in this zone. As for down reserve, it is the same as up reserve with two differences: All resources in zone *z* provide a down reserve, and contingency is not required. For reference case (without institutional barriers), operating reserve requirements are met at the regional level. We compute the contingency of the region as the maximum of the following three parameters: (i) the largest unit size of existing thermal resources, (ii) the largest capacity of power lines, and (iii) the largest unit size of candidate thermal resources.

Planning reserve: For coordination heterogeneity, installed capacity in each zone should exceed peak demand with a margin. We take into account fixed import and export as well. For reference case (without institutional barriers), planning reserve is met region-wide. Installed capacity region-wide should exceed regional peak demand with a margin. We remove fixed imports and exports.

Hurdle rate costs: For coordination heterogeneity, flows on lines are bi-directional, but hurdle rate costs are asymmetric in our case (e.g., the same amount of power is charged differently when it flows from Arizona to San Diego and from San Diego to Arizona). Hurdle rate costs are zero for zones within California. For reference case (without institutional barriers), hurdle rate costs are equal to 0.

CES constraints: Total annual dispatch from CES-eligible resources is larger than or equal to total annual dispatch from all resources, excluding storage units.

2.2. Objective function

We add the following in the objective function:

- Investment costs for all new build resources and fixed O&M costs for all resources
- Investment and fixed O&M costs per MWh for storage units
- Investment and fixed O&M costs for all transmission paths
- Total variable costs from all generators: The total variable cost of a generator ($/MWh) is variable O&M cost ($/MWh) plus total fuel cost ($/MWh). Total fuel cost is fuel cost ($/MMBtu) multiplied by heat rate (MMBtu/MWh).
- Cost of non-served demand
- Total start cost from all thermal plants: The total start cost of a thermal plant ($/MW) is the start cost ($/MW) plus the total fuel cost resulting from the start ($/MW). Total fuel cost is fuel cost ($/MMBtu) multiplied by fuel amount burned during the start operation (MMBtu/MW).
- Additionally, for reference case (without institutional barriers), we add hurdle rate costs.

2.3. Input data

We use generator and transmission line data, estimated demand for electricity in 2050, renewable energy profiles, and fuel prices for the western U.S. from PowerGenome (6). Detailed operational requirements come from RESOLVE (e.g., min/max dispatch bounds for reservoir hydro units) (7), SWITCH input data (e.g., operating reserve requirements) (8), and other data sources, see Tables S.2.2 - S.2.5.

Table S.2.2. Potentials (MW) for new build geothermal resources. Sources: (9, 10)

Zones	Potentials	Zones	Potentials	Zones	Potentials
Idaho	300	Nevada	1,400	North California	671
Pacific Northwest	800	San Diego	671	South California	671
Utah	300				

Table S.2.3. Potentials (MW) for new build non-powered dams. Source: (11)

Zones	Potentials	Zones	Potentials	Zones	Potentials
Arizona	80	Colorado	172	Idaho	12
Montana	88	New Mexico	103	Nevada	16
North California	81	Pacific Northwest	201	San Diego	82
South California	26	Utah	40	Wyoming	45

Table S.2.4. Potentials (MW) for new build pumped hydroelectric storage. Sources: (9, 10)

Zones	Potentials	Zones	Potentials	Zones	Potentials
Arizona	4,440	Colorado	790	Idaho	1,150
Montana	1,430	New Mexico	750	Nevada	2,300
North California	1,333	Pacific Northwest	4,898	San Diego	1,333
South California	1,333	Utah	3,100	Wyoming	1,850

Table S.2.5. Potentials (MW) for new build non-utility solar projects. Source: (12)

**Zones**	**Residential PV**	**Commercial PV**	**Solar Thermal with Energy Storage**
Arizona	7,500	7,500	3,528
Colorado	6,000	6,000	3,098
Idaho	1,500	1,500	1,267
Montana	1,000	1,000	557
New Mexico	2,000	2,000	4,860
Nevada	3,500	3,500	2,558
North California	12,666	12,666	908
Pacific Northwest	10,500	10,500	1076
San Diego	12,666	12,666	908
South California	12,666	12,666	908
Utah	3,000	3,000	1,638
Wyoming	500	500	1,956

3. Agent-based modeling (ABM)

The technical ODD description (13) and the code of the agent-based model can be found at https://github.com/lizverbeek/Energy_ABM. For reproducibility, here we provide a very brief description of the ABM, the conceptual model flow and an overview of the parameters used in the simulations presented in the paper.

Our agent-based model was developed to quantify aggregated consequences of individual behavioral changes, such as decisions to invest in solar panels, in the presence or not of informal social institutions. For the latter, following the classification of (14) we focus on socio-cognitive and normative institutions. Specifically, the reference scenario in the ABM model implements household decision-making as rational meaning that a household agent will pursue an action when it is economically-efficient. To represent the influence of socio-cognitive institutions, we add another layer of decision-making to the household agents in our ABM: an attitude towards the behavior parameterized using household survey data (15). Furthermore, to represent the influence of normative institutions, we rely on the survey responses that elicit the relative influence of social norms on individual behavior compared to own attitudes and financial considerations when deciding to install PVs. Besides the sensitivity analysis on social norms' effect, we also ran two scenarios: assuming that the influence of social norms remains static based on the survey data, or that it dynamically updated attitudes towards installing PV panels.

Figure S.3.1 shows the model flow for the household-decision making modules of these three implementations. Each of these decision-making processes are subject to budget constraints, i.e. a monetary barrier checking whether a household has enough savings to afford PV panels. Each household then compares the utility of installing PV panels to the utility of no action, and chooses the option with the highest utility. In the first implementation (Figure S.3.1.a), household decision-making is assumed to be rational, based solely on financial considerations regarding the installation of PV panels, i.e. households install PVs as soon as it appears economically-efficient. Since households vary in their energy costs and incomes/savings, they may decide to install PVs at different time steps (i.e. when they accumulate enough savings) or not at all. Second, the decision-making process is extended to account for informal institutions (Figure S.3.1.b). Here the individual households' considerations of costs and benefits are weighted in the utility function to account for attitudes and social norms regarding PV panels. Finally, the third implementation represents evolving informal institutions (Figure S.3.1.c), in which household decision-making is based on the attitude, social norm and perceived behavioral control (PBC), but here, attitudes are dynamically updated based on the attitudes of social connections. For a more detailed

description of these opinion dynamics, please also see the full ODD description of the model available online.

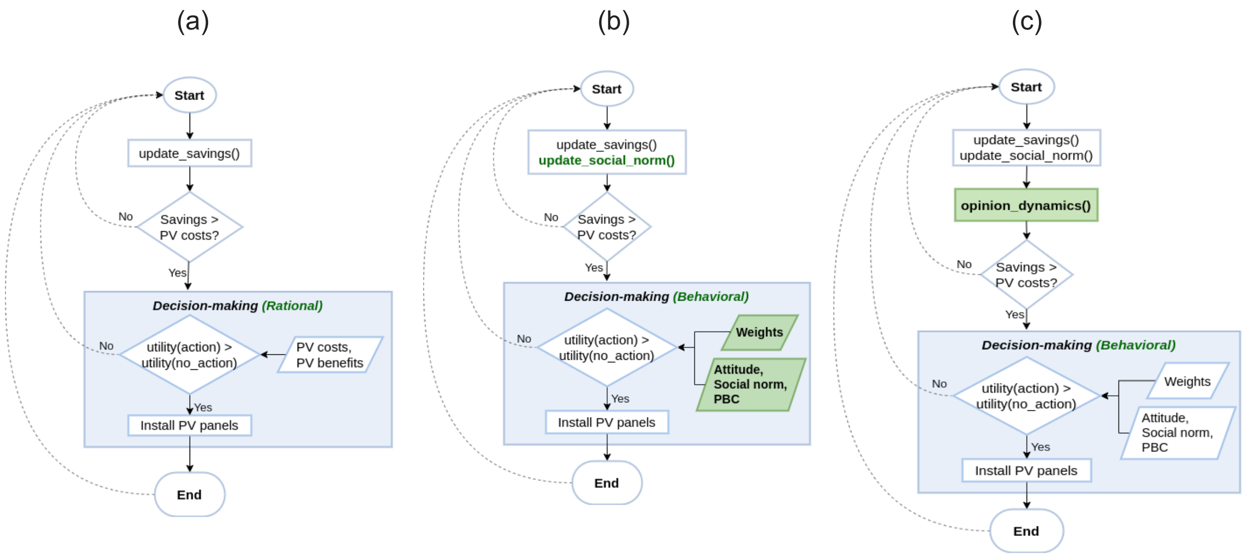

Figure S.3.1. Flow chart of the decision-making process of households in the agent-based model for each of the three implementations: (a) rational decision-making without the influence of informal institutions; (b) household's behavioral in the presence of static socio-cognitive (here only attitudes and perceived behavioral control, PBC) and normative (here only social norm) institutions; (c) household's behavior in the presence of dynamically updated attitudes towards installing solar panels.

The initial values for the household attributes employed for the simulations presented in the Main text come either from the household survey or secondary literature (Table S.3.1). These attributes are randomly drawn from empirical distributions based on previously collected empirical survey data from the Netherlands (15). In addition, constants used in the model include information on energy prices, estimated PV panel lifetime and peak power, and CO_2 emissions, are listed in Table S.3.2. For more detailed information, see the full ODD description of the model.

Table S.3.1. Initial household attributes of the agent-based energy model.

Attribute	Description	Distribution, (mean, st.dev.)	Range, (min; max)	Source
Income	Annual income (Euros)	$N(47052,\ 25232)$	$[3264,\ \infty]$	Survey, (15)
Energy use	Annual energy consumption	$N(2770,\ 1553)$	$[0,\ \infty]$	

Attribute	Description	Distribution, (mean, st.dev.)	Range, (min; max)	Source
	(kWh/year)			
Attitude	Attitude towards installing PVs	$N(0.2004,\ 0.4580)$	$[-1,\ 1]$	
w_{PBC}	TPB perceived behavioral control weight	-	$[0,\ 1]$	
w_A	TPB attitude weight	-	$[0,\ 1]$	
w_{SN}	TPB Social norm weight	-	$[0,\ 1]$	
N_{soc}	Number of social network connections	$N(7,\ 1)$	$[0,\ \infty]$	(16)
$\mathbf{w}_{inf} = [w_1, ..., w_n]$	Influence weights per social network connection	$w_i \sim U(0,\ 1)$ with $\sum_{i=1}^{n} w_i = 1$	$[0,\ 1]$	(17)

Table S.3.2. Constants and model parameters for the agent-based energy model.

Name	Value	Source
Savings ratio	0.235	(18)
Interest rate	-0.54877	(19)
PV panel lifetime (years)	25	(20)
PV peak power (Watt-peak (Wp))	370	(21)
PV panel efficiency loss	0.1	(20)
Electricity price (fixed cost) (Euros)	-228.70	(22)
Electricity price (variable cost) (Euros)	0.25618	(22)
CO_2 emission for electricity (kg/kWh)	0.425	(23)
Opinion influence rate (μ)	[0.01, 0.15]	Default value: drawn from the interval. See also the sensitivity analysis in Figure SI.2
Probability passing intention - action barrier (p_{barrier})	0.6	Assumption

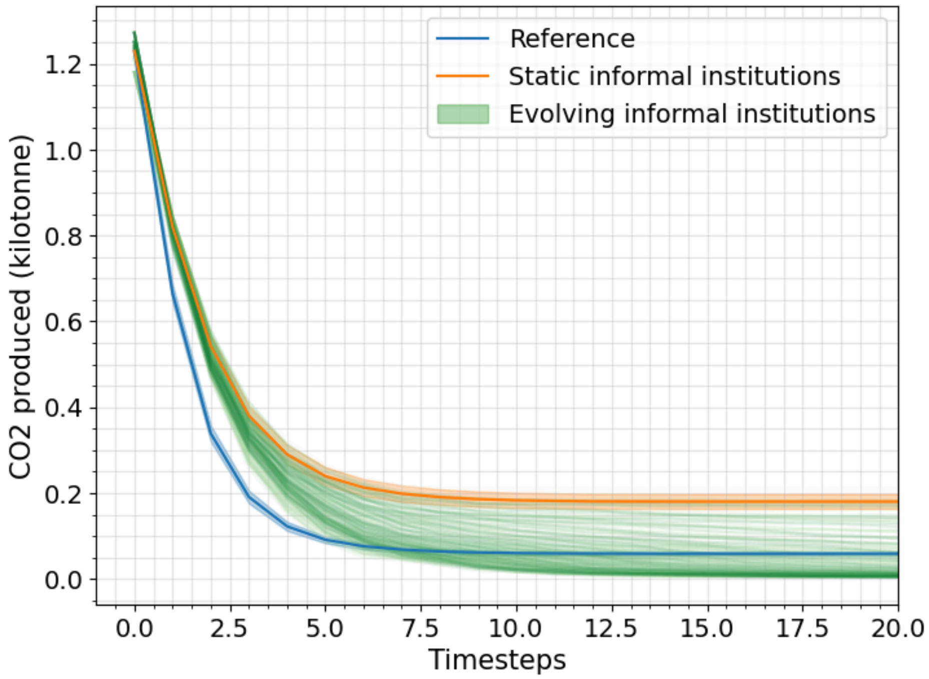

Figure S.3.2. Sensitivity analysis of the opinion dynamics influence rate (parameter μ) that impacts the speed and extent of dynamics of social norms, serving as a proxy of evolving informal institutions. Here μ is varied between 0.00 and 0.20 with intervals of 0.01.

To determine a suitable range for the influence rate (μ) that drives the opinion dynamics regarding households' attitudes, we performed sensitivity analysis of the model output under different values of this parameter. It is expected that a higher μ value results in a stronger effect on the uptake of PV panels, negative or positive. Since on average, attitude towards installing PV panels among Dutch households, as parameterized by the survey data, turned out to be positive (see Table S.3.1), it is expected that higher μ values result in a higher uptake of PV panels, and thus more reduction of CO_2 emissions. As shown in Figure S.3.2, this is indeed the case. Initially, μ values ranging from 0 to 0.2 are tested, with a value of 0 being equal to the model implementation without opinion dynamics (i.e. static informal institutions). For a lack of any well-informed value estimation for the influence of opinion dynamics rate parameter, in the final experiments, μ is varied between 0.1 and 0.15 over runs. With the lack of data on the speed of change in the attitudes via social interactions and opinion dynamics, which is reported to be important but still lacks data, we ran extensive sensitivity analysis to indicate how this simplistic representation of the evolution of informal institutional factors affect decarbonization transitions (Figure S.3.2).

References

1. K. Calvin, *et al.*, GCAM v5. 1: representing the linkages between energy, water, land, climate, and economic systems. *Geoscientific Model Development* **12**, 677–698 (2019).
2. W. Peng, *et al.*, Dataset for Peng et al. The Surprisingly Inexpensive Cost of State-Driven Emission Control Strategies. Nature Climate Change (2021). (2021) https:/doi.org/10.5281/zenodo.5061357 (November 20, 2022).
3. J. F. Clarke, J. A. Edmonds, Modelling energy technologies in a competitive market. *Energy Economics* **15**, 123–129 (1993).
4. A. A. Fawcett, *et al.*, Can Paris pledges avert severe climate change? *Science* **350**, 1168–1169 (2015).
5. P. D. Howe, M. Mildenberger, J. R. Marlon, A. Leiserowitz, Geographic variation in opinions on climate change at state and local scales in the USA. *Nature climate change* **5**, 596–603 (2015).
6. G. Schivley, *et al.*, PowerGenome (2022) (May 18, 2022).
7. CEC, "2021 SB 100 Joint Agency Report" (California Energy Commission, 2021).
8. J. Johnston, R. Henriquez-Auba, B. Maluenda, M. Fripp, Switch 2.0: A modern platform for planning high-renewable power systems. *SoftwareX* **10**, 100251 (2019).
9. B. Brownlee, *et al.*, "Western Flexibility Assessment Investigating the West's Changing Resource Mix and Implications for System Flexibility" (Energy Strategies, 2019).
10. CEC, Input & Assumptions - CEC SB 100 Joint Agency Report (2020).
11. B. Hadjerioua, Y. Wei, S.-C. Kao, "An Assessment of Energy Potential at Non-Powered Dams in the United States" (Oak Ridge National Laboratory, 2012).
12. A. Lopez, B. Roberts, D. Heimiller, N. Blair, G. Porro, "U.S. Renewable Energy Technical Potentials: A GIS-Based Analysis" (National Renewable Energy Laboratory, 2012).
13. V. Grimm, *et al.*, The ODD Protocol for Describing Agent-Based and Other Simulation Models: A Second Update to Improve Clarity, Replication, and Structural Realism. *JASSS* **23**, 7 (2020).
14. W. R. Scott, *Institutions and organizations: Ideas, interests, and identities* (SAGE Publications, 2013).
15. L. Niamir, O. Ivanova, T. Filatova, A. Voinov, H. Bressers, Demand-side solutions for climate mitigation: Bottom-up drivers of household energy behavior change in the Netherlands and Spain. *Energy Research & Social Science* **62**, 101356 (2020).
16. A. Taberna, T. Filatova, A. Roventini, F. Lamperti, Coping with increasing tides: Evolving agglomeration dynamics and technological change under exacerbating hazards. *Ecological Economics* **202**, 107588 (2022).
17. A. Flache, *et al.*, Models of Social Influence: Towards the Next Frontiers. *JASSS* **20**, 2 (2017).
18. Centraal Bureau voor de Statistiek, Key figures by sector; National Accounts (2022) (July 22, 2022).
19. De Nederlandsche Bank, Interest rates (2022) (July 27, 2022).
20. Consumentenbond, Terugverdientijd zonnepanelen (2022) (July 25, 2022).
21. Milieu Centraal, Kosten en opbrengst zonnepanelen (2022) (July 25, 2022).
22. Centraal Bureau voor de Statistiek, Average energy prices for consumers (2022) (July 22, 2022).
23. Centraal Bureau voor de Statistiek, Rendementen, CO2-emissie elektriciteitsproductie (2022) (July 22, 2022).
